# Multicenter Evaluation of the Cepheid Xpert Xpress SARS-CoV-2/Flu/RSV Test

**DOI:** 10.1128/JCM.02955-20

**Published:** 2021-02-18

**Authors:** Heba H. Mostafa, Karen C. Carroll, Rachel Hicken, Gregory J. Berry, Ryhana Manji, Elizabeth Smith, Jennifer L. Rakeman, Randal C. Fowler, Mindy Leelawong, Susan M. Butler-Wu, David Quintero, Minette Umali-Wilcox, Robert W. Kwiatkowski, David H. Persing, Fred Weir, Michael J. Loeffelholz

**Affiliations:** a The Johns Hopkins University School of Medicine, Baltimore, Maryland, USA; b Northwell Health Laboratories, Zucker SOM at Hofstra/Northwell, East Garden City, New York, USA; c NYC Public Health Laboratory, Department of Health and Mental Hygiene, New York, New York, USA; d Keck School of Medicine of USC, Department of Pathology, Los Angeles, California, USA; e Cepheid, Sunnyvale, California, USA; Boston Children’s Hospital

**Keywords:** Cepheid, SARS-CoV-2, influenza, flu, RSV, 4-plex

## Abstract

With the approach of respiratory virus season in the Northern Hemisphere, clinical microbiology and public health laboratories will need rapid diagnostic assays to distinguish severe acute respiratory syndrome coronavirus 2 (SARS-CoV-2) from influenza virus and respiratory syncytial virus (RSV) infections for diagnosis and surveillance. In this study, the clinical performance of the Xpert Xpress SARS-CoV-2/Flu/RSV test (Cepheid, Sunnyvale, CA, USA) for nasopharyngeal swab specimens was evaluated in four centers: Johns Hopkins Medical Microbiology Laboratory, Northwell Health Laboratories, NYC Public Health Laboratory, and Los Angeles County/University of Southern California (LAC+USC) Medical Center. A total of 319 nasopharyngeal swab specimens, positive for SARS-CoV-2 (*n* = 75), influenza A virus (*n* = 65), influenza B virus (*n* = 50), or RSV (*n* = 38) or negative (*n* = 91) by the standard-of-care nucleic acid amplification tests at each site, were tested using the Cepheid panel test. The overall positive percent agreement for the SARS-CoV-2 target was 98.7% (*n* = 74/75), and the negative agreement was 100% (*n* = 91), with all other analytes showing 100% total agreement (*n* = 153). Standard-of-care tests to which the Cepheid panel was compared included the Cepheid Xpert Xpress SARS-CoV-2, Cepheid Xpert Xpress Flu/RSV, GenMark ePlex respiratory panel, BioFire respiratory panel 2.1 and v1.7, DiaSorin Simplexa COVID-19 Direct, and Hologic Panther Fusion SARS-CoV-2 assays. The Xpert Xpress SARS-CoV-2/Flu/RSV test showed high sensitivity and accuracy for all analytes included in the test. This test will provide a valuable clinical diagnostic and public health solution for detecting and differentiating SARS-CoV-2, influenza A and B virus, and RSV infections during the current respiratory virus season.

## Abstract (precis)

With the approach of respiratory virus season in the Northern Hemisphere, clinical microbiology and public health laboratories will need rapid diagnostic assays to distinguish severe acute respiratory syndrome coronavirus 2 (SARS-CoV-2) from influenza virus and respiratory syncytial virus (RSV) infections for diagnosis and surveillance. In this study, the clinical performance of the Xpert Xpress SARS-CoV-2/Flu/RSV test (Cepheid, Sunnyvale, CA, USA) for nasopharyngeal swab specimens was evaluated in four centers: Johns Hopkins Medical Microbiology Laboratory, Northwell Health Laboratories, NYC Public Health Laboratory, and Los Angeles County/University of Southern California (LAC+USC) Medical Center.

## INTRODUCTION

The potential concurrent circulation of severe acute respiratory syndrome coronavirus 2 (SARS-CoV-2), influenza viruses, and respiratory syncytial virus (RSV) may prove to be a challenge for health care providers and clinical microbiology and public health laboratories. The ability to differentiate the diseases caused by these viruses is essential for patient management and infection control, as well as public health surveillance and responses. These viruses can cause infections that present with very similar symptoms, making clinical differentiation between them very difficult (1). Clinical microbiology and public health laboratories are likely to face pressure to offer parallel testing for these viruses, optimally using rapid assays with the simultaneous detection of SARS-CoV-2, influenza A and B viruses, and RSV. Critically, positivity for one target does not rule out infection with another respiratory virus, with coinfection with SARS-CoV-2 and influenza virus as well as other respiratory viruses reported (2–7). Therefore, an optimal diagnostic algorithm for testing patients with influenza-like disease is a multiplex assay that combines the four targets to simultaneously test for SARS-CoV-2, influenza A virus, influenza B virus, and RSV.

The Xpert Xpress SARS-CoV-2/Flu/RSV test (Cepheid, Sunnyvale, CA, USA) is a multiplexed rapid real-time reverse transcriptase PCR (rRT-PCR) that can detect and differentiate SARS-CoV-2, influenza A virus, influenza B virus, and RSV. This test is the first and only test thus far that detects all four viruses in a single quadriplex panel to receive emergency-use authorization (EUA) from the U.S. Food and Drug Administration (FDA). The test is a closed test that integrates specimen extraction, reverse transcription, amplification, and target detection with minimal hands-on time and an ∼36-min time to results. In this study, we describe the performance of the test in four different laboratories.

## MATERIALS AND METHODS

### The Xpert Xpress SARS-CoV-2/Flu/RSV test.

The test received FDA EUA for viral RNA detection in upper respiratory tract specimens, which include nasopharyngeal or nasal swabs and nasal wash specimens/aspirates. The test cartridge is run on the GeneXpert Dx, GeneXpert Xpress, or GeneXpert Infinity system, and result interpretation is performed by the instrument software. The test detects targets in the SARS-CoV-2 genes E and N2, similar to the Xpert Xpress SARS-CoV-2 test (8), but in contrast to the latter, the targets are combined in the same optical detection channel, and hence, the test does not provide separate results for each of the two targets. Details of the analytical evaluation of the assay, including the analytical sensitivity, are available in the most updated assay’s package insert (https://www.fda.gov/media/142437/download). Research-use-only (RUO) cartridges were distributed to four different sites to compare the performance of the Xpert Xpress SARS-CoV-2/Flu/RSV test to those of the standard-of-care (SOC) SARS-CoV-2, influenza A virus, influenza B virus, and RSV tests. Each site tested archived specimens with the SOC assay in use at the site as the comparators.

### Specimens and standard-of-care testing.

Table 1 lists the participating sites and their standard-of-care test methods. For all sites, specimens were collected from both symptomatic and asymptomatic patients of all age groups. Of note, no coinfections with SARS-CoV-2 and influenza A or B virus or RSV were noted in the tested cohorts at all sites.

**TABLE 1:** Specimens and standard-of-care testing for each study site^*a*^

Study site	Specimen source	No. of specimens tested/standard-of-care test(s)				
		Positive for SARS-CoV-2	Positive for influenza A virus	Positive for influenza B virus	Positive for RSV	Negative
Northwell Health Laboratories	NPS in UTM	16/Panther Fusion SARS-CoV-2 assay^*b*^	10/BioFire RP2.1^*d*^	10/BioFire RP2.1^*d*^	10/BioFire RP2.1^*d*^	30/BioFire RP2.1^*c*^^,^^*d*^
New York City Public Health Laboratory	NPS in VTM	20/Xpert Xpress SARS-CoV-2	20/CDC human influenza virus real-time RT-PCR diagnostic panel	20/CDC human influenza virus real-time RT-PCR diagnostic panel	0	20/Xpert Xpress SARS-CoV-2/BioFire respiratory panel v1.7
Johns Hopkins Hospital	NPS in VTM	20/Xpert Xpress SARS-CoV-2	15 (11/Xpert Xpress Flu/RSV and 4/GenMark ePlex respiratory pathogen panel)	10 (6/Xpert Xpress Flu/RSV and 4/GenMark ePlex respiratory pathogen panel)	10 (7/Xpert Xpress Flu/RSV and 3/GenMark ePlex respiratory pathogen panel)	20/Xpert Xpress SARS-CoV-2
LAC+USC Medical Center	NPS in VTM/UTM	19/Xpert Xpress SARS-CoV-2	20/Xpert Xpress Flu/RSV	10/Xpert Xpress Flu/RSV	18/Xpert Xpress Flu/RSV	21/Xpert Xpress SARS-CoV-2^*e*^

a NPS, nasopharyngeal swab; UTM, universal transport medium; VTM, viral transport medium. RP2.1, respiratory panel 2.1.

b Specimens were also tested by BioFire RP2.1.

c Twenty specimens were positive for targets other than SARS-CoV-2, influenza A virus, influenza B virus, and RSV.

d Specimens were also tested by the GenMark ePlex respiratory pathogen panel.

e Six specimens were also tested by the DiaSorin COVID-19 Direct assay.

### (i) Northwell Health Laboratories.

Nasopharyngeal swab (NPS) specimens (*n* = 76) were initially collected in 3 ml universal transport medium (UTM) (various manufacturers). The Panther Fusion SARS-CoV-2 assay was used as the SOC test for the detection of SARS-CoV-2 (https://www.fda.gov/media/136156/download), and the GenMark ePlex respiratory pathogen panel was the SOC for influenza A virus, influenza B virus, and RSV targets (https://www.accessdata.fda.gov/cdrh_docs/pdf16/K163636.pdf). Residual specimens were immediately aliquoted and frozen at −80°C, remaining frozen until this study was performed. Samples were thawed and immediately tested by the Xpert Xpress SARS-CoV-2/Flu/RSV test. In addition, side-by-side testing at the same time with BioFire respiratory panel 2.1 (RP2.1) was performed to collect comparative data for the two assays as well (https://www.fda.gov/media/137583/download). Notably, 20 specimens were positive for other targets on the BioFire panel, which included adenovirus (*n* = 1), Chlamydia pneumoniae (*n* = 1), endemic coronavirus (*n* = 10), enterovirus/rhinovirus (*n* = 1), human metapneumovirus (*n* = 1), Mycoplasma pneumoniae (*n* = 1), and human parainfluenza virus (*n* = 5). Those 20 specimens were considered negative for SARS-CoV-2, influenza A virus, influenza B virus, and RSV.

### (ii) NYC Public Health Laboratory, Department of Health and Mental Hygiene.

Nasopharyngeal swab specimens (*n* = 80) collected in viral transport medium (VTM) were initially tested by the standard-of-care CDC human influenza virus real-time RT-PCR diagnostic panel for influenza A virus and influenza B virus or the Xpert Xpress SARS-CoV-2 test for SARS-CoV-2 (https://www.fda.gov/media/136314/download). Negative nasopharyngeal swab specimens were preliminarily identified by a negative result with the Xpert Xpress SARS-CoV-2 test and were confirmed negative for influenza A/B virus and RSV by BioFire FilmArray respiratory panel v1.7 prior to testing by the Xpert Xpress SARS-CoV-2/Flu/RSV test. Specimens were stored at −70°C until Xpert Xpress SARS-CoV-2/Flu/RSV analysis.

### (iii) The Johns Hopkins Hospital Laboratory.

Nasopharyngeal swab specimens (*n* = 75) were collected in VTM from 45 adult and 30 pediatric patients (<18 years of age). SOC testing was performed by the Xpert Xpress SARS-CoV-2 test, the Xpert Xpress Flu/RSV test, and the GenMark ePlex respiratory pathogen panel assay. Residual specimens were aliquoted and frozen at −70°C until tested by the Xpert Xpress SARS-CoV-2/Flu/RSV test for the current study.

### (iv) Los Angeles County/University of Southern California (LAC+USC) Medical Center.

Nasopharyngeal swab specimens (*n* = 88) were collected in VTM or UTM (various manufacturers). Initial standard-of-care testing was performed using the Xpert Xpress SARS-CoV-2 test, the Xpert Xpress Flu/RSV test, and the Simplexa COVID-19 Direct test (DiaSorin) (*n* = 6 specimens). Leftover specimens were stored at −70°C until the time of testing with the Xpert Xpress SARS-CoV-2/Flu/RSV panel test.

In addition to the qualitative result of RNA detected or RNA not detected, correlation of the cycle threshold (*C_T_*) values was made, where available, between assays.

### Ethical concerns.

The study protocol was reviewed and approved by the institutional review boards at the study testing sites or conducted as a quality improvement project consistent with institutional policies.

### Statistical analysis.

Positive and negative percent agreements for the Xpert Xpress SARS-CoV-2/Flu/RSV test were calculated using two-by-two tables. Overall accuracy, positive percent agreement, negative percent agreement, and 95% confidence intervals (CIs) were calculated using MedCalc statistical software version 19.2.6 (MedCalc Software Ltd., Ostend, Belgium). Linear regression analysis was performed using GraphPad Prism for comparing the *C_T_* values of different assays.

## RESULTS

### Agreement of the Xpert Xpress SARS-CoV-2/Flu/RSV test with comparator standard-of-care methods.

**FIG 1:**
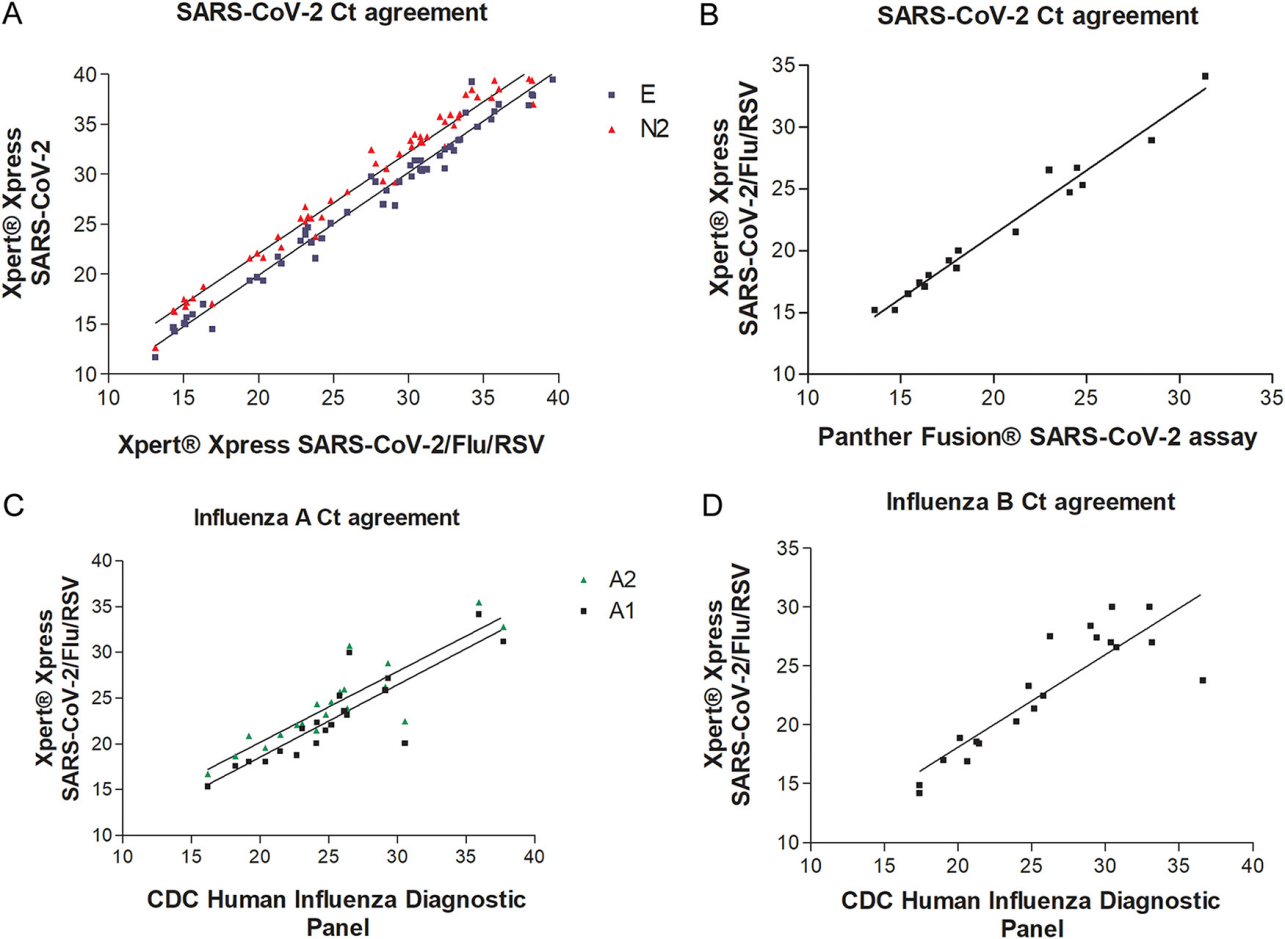
Correlation of the Cepheid Xpert Xpress SARS-CoV-2/Flu/RSV test cycle threshold (*C_T_*) values with the standard-of-care test *C_T_* values for SARS-CoV-2 (A and B), influenza A virus (C), and influenza B virus (D). Notably, The Cepheid Xpert Xpress SARS-CoV-2/Flu/RSV test detects SARS-CoV-2 genes E and N2, similar to the Xpert Xpress SARS-CoV-2 test, but in contrast, the two targets are not differentiated (A and B).

A total of 166 nasopharyngeal swab specimens initially tested by the SOC assays for SARS-CoV-2 were tested by the Xpert Xpress SARS-CoV-2/Flu/RSV test. The Xpert Xpress SARS-CoV-2/Flu/RSV test missed only one positive specimen (initially positive with Xpert Xpress SARS-CoV-2 with a *C_T_* value of E of 41.1 and a *C_T_* value of N2 of 43.3). The overall agreement was 99.3% (*n* = 165/166), with a positive agreement of 98.7% (*n* = 74/75) (95% CI of 91.02 to 99.8%) and a negative agreement of 100% (*n* = 91/91) (Table 2). Notably, two Xpert Xpress SARS-CoV-2-positive specimens had a single *C_T_* value of N2 of 41.5 or 39.2 and a negative E target (Fig. 1A). The Xpert Xpress SARS-CoV-2/Flu/RSV test showed excellent agreement for *C_T_* values with both the Xpert Xpress SARS-CoV-2 (for the E gene, a slope of 1.027 ± 0.02 and a *y* intercept of −0.59 ± 0.6 [*R*^2^ = 0.97]; for the N2 gene, a slope of 1.01 ± 0.02 and a *y* intercept of 1.9 ± 0.7 [*R*^2^ = 0.97]) and Panther Fusion SARS-CoV-2 (for the open reading frame 1ab [ORF1ab] gene, a slope of 1.03 ± 0.04 and a *y* intercept of 0.6 ± 0.9 [*R*^2^ = 0.97]) tests (Fig. 1A and B).

**TABLE 2:** Agreement of the Xpert Xpress SARS-CoV-2/Flu/RSV test with the comparator standard-of-care SARS-CoV-2 tests^*a*^

Test(s)	No. of specimens with Cepheid Xpert Xpress SARS-CoV-2/Flu/RSV test result/comparator test result (SARS-CoV-2) of:				PPA (95% CI)	NPA
	Pos/Pos	Pos/Neg	Neg/Pos	Neg/Neg		
All methods	74	0	1	91	98.7 (91.02–99.8)	100
Xpert Xpress SARS-CoV-2	58	0	1	41		
Panther Fusion SARS-CoV-2 assay	16	NA	0	NA		
BioFire RP2.1	NA	0	NA	30		
BioFire respiratory panel v1.7	NA	0	NA	20		

a Pos, positive; Neg, negative; PPA, positive percent agreement; NPA, negative percent agreement; CI, confidence interval; NA, not applicable.

The agreement of the Xpert Xpress SARS-CoV-2/Flu/RSV test with the SOC influenza A virus/influenza B virus/RSV tests at the four sites was 100%. A total of 65 influenza A virus-, 50 influenza B virus-, and 38 RSV-positive NPS specimens as well as 50 negative specimens (notably, the negative specimens were a subset of the 91 SARS-CoV-2-negative samples [Table 2] that were confirmed to be negative for influenza A virus, influenza B virus, and RSV before the study) were tested, and the results were compared to those of the SOC tests (Table 3). The performance was comparable to those of the Xpert Xpress Flu/RSV test, BioFire respiratory panel 2.1 (RP2.1), the GenMark ePlex respiratory pathogen panel, and the CDC human influenza virus real-time RT-PCR diagnostic panel. Notably, the Xpert Xpress SARS-CoV-2/Flu/RSV test showed good correlation at the *C_T_* value level with the CDC human influenza virus real-time RT-PCR diagnostic panel for both influenza A virus (for Cepheid channel A1, a slope of 0.8 ± 0.1 and a *y* intercept of 2.7 ± 2.8 [*R*^2^ = 0.75]; for Cepheid channel A2, a slope of 0.8 ± 0.1 and a *y* intercept of 4.7 ± 2.4 [*R*^2^ = 0.79]) and influenza B virus (a slope of 0.8 ± 0.1 and a *y* intercept of 2.4 ± 2.8 [*R*^2^ = 0.76]) (Fig. 1C and D).

**TABLE 3:** Agreement of the Xpert Xpress SARS-CoV-2/Flu/RSV test with the comparator standard-of-care influenza A virus, influenza B virus, and RSV tests^*a*^

Test(s)	No. of specimens with Cepheid Xpert Xpress SARS-CoV-2/Flu/RSV test result/comparator test result of:			
	Pos/Pos	Pos/Neg	Neg/Pos	Neg/Neg
Influenza A virus				
All methods	65	0	0	50
BioFire RP2.1	10	0	0	30
Xpert Xpress Flu/RSV	31	NA	0	NA
GenMark ePlex respiratory pathogen panel	4	NA	0	NA
CDC human influenza virus real-time RT-PCR diagnostic panel	20	NA	0	NA
BioFire respiratory panel v1.7	NA	0	NA	20

Influenza B virus				
All methods	50	0	0	50
BioFire RP2.1	10	0	0	30
Xpert Xpress Flu/RSV	16	NA	0	NA
GenMark ePlex respiratory pathogen panel	4	NA	0	NA
CDC human influenza virus real-time RT-PCR diagnostic panel	20	NA	0	NA
BioFire respiratory panel v1.7	NA	0	NA	20

RSV				
All methods	38	0	0	50
BioFire RP2.1	10	0	0	30
Xpert Xpress Flu/RSV	25	NA	0	NA
GenMark ePlex respiratory pathogen panel	3	NA	0	NA
BioFire respiratory panel v1.7	NA	0	NA	20

a Pos, positive; Neg, negative; NA, not applicable.

## DISCUSSION

The SARS-CoV-2 pandemic will impact algorithms for influenza-like illness (ILI) testing. In a typical influenza season, molecular testing for influenza virus is recommended if the patient is likely to be hospitalized or if a conclusive influenza diagnosis could impact the patient’s management (9). Testing for additional respiratory viral pathogens that can cause influenza-like illness, such as RSV, human parainfluenza viruses, enteroviruses/rhinoviruses, and human metapneumoviruses, among others, may be warranted in pediatric, elderly, and immunocompromised patients (10). Offering SARS-CoV-2 testing along with influenza virus testing for symptomatic patients will be essential with the start of the 2020 to 2021 influenza season in the Northern Hemisphere driven by the widespread community prevalence of SARS-CoV-2 and the very similar clinical presentations. Differentiation of COVID-19 (coronavirus disease caused by SARS-CoV-2) from influenza and other respiratory diseases in hospitalized and nonhospitalized patients will also be critical for public health surveillance and responses to the ongoing pandemic.

In response to the COVID-19 global pandemic, a growing number of molecular diagnostic assays have become commercially available; assays differ in detection targets as well as analytical sensitivity, and most demonstrate high specificity (11–29). Variables that contribute to assay performance include the type of specimen examined, the time of specimen collection in relation to the course of illness, as well as the adequacy of specimen collection (30–34). The Xpert Xpress SARS-CoV-2 test received FDA EUA on 20 March 2020 and was the first assay authorized for use in Clinical and Laboratory Improvement Amendments (CLIA)-waived settings. The use of this sensitive test in the laboratory and point-of-care settings (8, 28, 35, 36) has reduced health care workers’ exposure risk in emergency rooms due to the short turnaround time (37).

The laboratory diagnosis of influenza during the 2020 to 2021 flu season is of particular importance due to the circulation of SARS-CoV-2, overlapping clinical presentations of both influenza and COVID-19, as well as distinct infection control and public health ramifications of the two viruses in the setting of the global COVID-19 pandemic. Testing all patients presenting with ILI/COVID-19-like illness for relevant circulating respiratory viruses will be critical to the diagnosis and appropriate care of the patient, to identify coinfections, and to initiate appropriate public health interventions and collect accurate surveillance data. In addition, RSV detection is particularly valuable due to the seasonal overlap of influenza and similar symptoms in some patient populations, including pediatric and immunocompromised patients. The Cepheid Xpress Flu/RSV test is one of a few rapid molecular assays available for influenza virus and RSV detection. The test, since its implementation in microbiology laboratories or in CLIA-waived settings, showed high sensitivity and specificity for the detection of influenza A and B viruses and RSV and a positive clinical impact associated with the rapid availability of the results (38–42).

A limited number of commercial molecular assays, as of the writing of the manuscript, in addition to the Cepheid Xpert Xpress SARS-CoV-2/Flu/RSV test have combined SARS-CoV-2 testing with testing for other viruses such as influenza virus or RSV. These tests include small panels developed by the CDC (influenza SARS-CoV-2 [Flu SC2] multiplex assay) and Roche Molecular Systems (Cobas SARS-CoV-2 & Influenza A/B) for the Cobas and Liat systems. In addition to these small panels, extended panels from BioFire Diagnostics LLC (BioFire respiratory panel 2.1 [RP2.1] and RP2.1-EZ), Qiagen (QIAstat-Dx respiratory SARS-CoV-2 panel), and GenMark (ePlex respiratory pathogen panel 2 [RP2]) are available. The Cepheid Xpert Xpress SARS-CoV-2/Flu/RSV test is currently the only commercially available quadriplex panel that combines the detection of SARS-CoV-2, influenza A and influenza B viruses, and RSV. The test has a short time to results and is available for CLIA-waived and high-throughput-format platforms. Our study showed that the Xpert Xpress SARS-CoV-2/Flu/RSV test has high sensitivity and accuracy for the four assay targets. As a result, implementing a multiplex SARS-CoV-2, influenza A and B virus, and RSV test is projected to have a positive impact during the respiratory virus season of 2020 to 2021 associated with upfront testing for SARS-CoV-2, influenza A and B viruses, and RSV and the short turnaround time.
